# The Molecular Identification and Antifungal Susceptibility of Clinical Isolates of *Aspergillus* Section *Flavi* from Three French Hospitals

**DOI:** 10.3390/microorganisms11102429

**Published:** 2023-09-28

**Authors:** Elie Djenontin, Jean-Marc Costa, Bita Mousavi, Lin Do Ngoc Nguyen, Jacques Guillot, Laurence Delhaes, Françoise Botterel, Eric Dannaoui

**Affiliations:** 1 Dynamyc UPEC, EnvA, USC Anses, Faculté de Médecine de Créteil, 94010 Créteil, France; elie.djenontinagossou@aphp.fr (E.D.); bimousavi@gmail.com (B.M.); francoise.botterel@aphp.fr (F.B.); 2 Service de Parasitologie-Mycologie, Hôpital Universitaire Mondor, AP-HP, 8 Rue du Général Sarrail, 94010 Créteil, France; 3 Laboratoire CERBA, 11 Rue de l’Équerre, 95310 Saint-Ouen-l’Aumône, France; jmcosta@lab-cerba.com; 4 Family Hospital, 73 Nguyen Huu Tho, Hai Chau, Danang 55000, Vietnam; nguyendongoclinh@gmail.com; 5 Unité pédagogique de Dermatologie, Parasitologie, Mycologie, Ecole Nationale Vétérinaire Agroalimentaire et de l’Alimentation Nantes Atlantique, Oniris, 44300 Nantes, France; jacques.guillot@oniris-nantes.fr; 6 Laboratoire de Parasitologie-Mycologie, CNR des Aspergilloses Chroniques—CHU de Bordeaux, INSERM U1045—Univ. Bordeaux, 33000 Bordeaux, France; laurence.delhaes@u-bordeaux.fr; 7 Faculté de Médecine, Université Paris Cité, 75006 Paris, France; 8 Unité de Parasitologie-Mycologie, Hôpital Necker Enfants Malades, AP-HP, 149 Rue de Sèvres, 75015 Paris, France

**Keywords:** *Flavi* section, *Aspergillus flavus*, *Aspergillus sojae*, *Aspergillus parasiticus*, *Aspergillus nomiae*, cryptic species, antifungal resistance

## Abstract

(1) Background: *Aspergillus flavus* is a cosmopolitan mold with medical, veterinary, and agronomic concerns. Its morphological similarity to other cryptic species of the *Flavi* section requires molecular identification techniques that are not routinely performed. For clinical isolates of *Aspergillus* section *Flavi*, we present the molecular identification, susceptibility to six antifungal agents, and clinical context of source patients. (2) Methods: One hundred forty fungal clinical isolates were included in the study. These isolates, recovered over a 15-year period (2001–2015), were identified based on their morphological characteristics as belonging to section *Flavi*. After the subculture, sequencing of a part of the β-tubulin and calmodulin genes was performed, and resistance to azole antifungals was screened on agar plates containing itraconazole and voriconazole. Minimum inhibitory concentrations were determined for 120 isolates by the European Committee on Antimicrobial Susceptibility Testing (EUCAST) broth microdilution method. (3) Results: Partial β-tubulin and calmodulin sequences analysis showed that 138/140 isolates were *A. flavus sensu stricto*, 1 isolate was *A. parasiticus/sojae,* and 1 was *A. nomiae*. Many of the isolates came from samples collected in the context of respiratory tract colonization. Among probable or proven aspergillosis, respiratory infections were the most frequent, followed by ENT infections. Antifungal susceptibility testing was available for isolates (n = 120, all *A. flavus ss*) from one hospital. The MIC range (geometric mean MIC) in mg/L was 0.5–8 (0.77), 0.5–8 (1.03), 0.125–2 (0.25), 0.03–2 (0.22), 0.25–8 (1.91), and 0.03–0.125 (0.061) for voriconazole, isavuconazole, itraconazole, posaconazole, amphotericin B, and caspofungin, respectively. Two (1.67%) isolates showed resistance to isavuconazole according to current EUCAST breakpoints with MICs at 8 mg/L for isavuconazole and voriconazole. One of these two isolates was also resistant to itraconazole with MIC at 2 mg/L. (4) Conclusions: The present characterization of a large collection of *Aspergillus* belonging to the *Flavi* section confirmed that *A. flavus ss* is the predominant species. It is mainly implicated in respiratory and ENT infections. The emergence of resistance highlights the need to perform susceptibility tests on section *Flavi* isolates.

## 1. Introduction

*A. flavus* is the second most common pathogenic species of *Aspergillus* in humans [1]. Indeed, *A. flavus* can be responsible for invasive aspergillosis (IA), chronic aspergillosis, and other infections [2]. *A. flavus* is the first cause of primary cutaneous aspergillosis [3,4]. Auricular and ocular localizations are frequently reported as keratitis and chronic otomycosis [5,6,7,8,9,10,11]. *A. flavus* is also the first agent of skull base aspergillosis, whose typical form is malignant external otitis [12,13,14,15,16,17], and the principal risk factor is diabetes [18]. *A. flavus* could also be implicated in many other clinical forms such as pulmonary invasive aspergillosis [19,20,21,22], chronic rhinosinusitis [23], brain abscesses [24], myositis [25,26], arthritis [27], spondylodiscitis [28,29], endocarditis [30,31], mediastinitis [32], eumycetoma [33], and allergic [34] or hypersensitivity syndromes like hot tub pneumonitis [35].

*A. flavus* is also of great importance due to its ability to cause disease in animals and crops and to produce carcinogenic mycotoxins [36]. Another epidemiological characteristic of *A. flavus* is its higher prevalence in tropical countries [37,38,39].

Section *Flavi* includes 35 species divided into eight series [40,41]. All these species share morphological characteristics, and some are morphologically indistinguishable. Nevertheless, whole-genome sequencing data have recently shown the genetic and metabolic diversity among the *Flavi* section [42]. Only *A. flavus ss*, *A. nomiae*, *A. minisclerotigenes, A. tamarii,* and *A. alliaceus* have been reported as pathogenic in humans [6,43,44,45].

Nevertheless, the prevalence of the different cryptic species in clinical samples is largely unknown, as is their pathogenicity. None of the cryptic species in section *Flavi* are known to carry natural resistance to antifungal drugs [46]. However, *A. flavus* shows high amphotericin B MICs compared to *A. fumigatus* [47].

The emergence of acquired azole resistance observed in the last 20 years in the genus *Aspergillus* has been mainly reported in *A. fumigatus* [48] but also affects other *Aspergillus* species [49,50,51,52]. The azole resistance of *A. flavus* clinical isolates has been reported since 2012 [53,54,55,56].

In this study, we molecularly analyze 140 isolates belonging to the *Flavi* section and determine the susceptibility profile for a subset (n = 120) of the isolates.

## 2. Materials and Methods

### 2.1. Study Design, Patients and Isolates

A total of 140 clinical isolates of *Aspergillus* section *Flavi* recovered from 107 patients were analyzed. Isolates were mainly cultured from respiratory samples (n = 114) and ENT sphere (n = 19). Seven isolates were cultured from other sites: two artery biopsies, dialysis fluid, nail, arm biopsy, hallux biopsy, and endocardial biopsy (Table S1). All isolates were collected between 2001 and 2015 through routine clinical work from patients of three hospitals: Hôpital Henri Mondor (HMN), Hôpital Universitaire de Lile (LIL), and Hôpital Européen Georges Pompidou (HEGP). The number of isolates collected each year was homogeneous over time, with 72 isolates between 2001 and 2008 and 68 isolates between 2009 and 2015. These isolates were stored frozen. Patients’ identifiable information had already been anonymized. Since the study was conducted on isolates collected through routine clinical work and patients’ identifiable information had already been anonymized, no written or verbal informed consent was necessary for patients to participate in this study. We testify that we followed the ethical standards of the Helsinki Declaration of 1975, as revised in 2008. Patients include lung transplant patients with cystic fibrosis and other pathologies, patients with hematological malignancies, and patients with ENT diseases and other pathologies. Clinical data showed that most of the isolates (96 isolates) were considered colonizers; 41 isolates were considered implicated in infections, including invasive pulmonary aspergillosis (12 isolates), otitis (12 isolates), bronchopulmonary aspergillosis (6 isolates), sinusitis (1 isolate), aspergilloma (1 isolate), endovascular infection (2 isolates), endocarditis (1 isolate), nasal aspergillosis (1 isolate), hallux infection (1 isolate), arm infection (1 isolate), and one disseminated invasive aspergillosis (1 isolate). For five isolates, clinical data were not available. 

### 2.2. Molecular Identification

DNA extraction: Molds were subcultured in Sabouraud medium for 7 days at 35 °C. Conidia were subjected to a mechanical shock with MagNA Lyser Green Beads (Roche Diagnostics, Meylan, France) in MagNA Lyser Instrument (Roche) and thermal shock in the ice to disrupt the wall. Then, after 4 h incubation at 56 °C with proteinase K (Qiagen Sciences Inc., Courtaboeuf, France), DNA was extracted using the QIAamp DNA Blood Mini Kit (Qiagen Sciences Inc.) following the manufacturer’s instructions.

Sequencing: Molecular identification was performed by partial sequencing of the β-tubulin and calmodulin genes using primers bt2a (5′-GGTAACCAAATCGGTGCTGCTTTC-3′); bt2b (5′-ACCCTCAGTGTAGTGACCCTTGGC-3′) designed by Glass and Donaldson [57] and cmd5 (5′-CCGAGTACAAGGARGCCTTC-3′); cmd6 (5′-CCGATRGAGGTCATRACGTGG-3′) designed by Hong et al. [58]. Primers were synthesized by Sigma-Aldrich (Saint Quentin-Fallavier, France). Each sample reaction mixture contained 4 µM (1 µL at 100 µM) of each primer; 2 mM MgCl_2_ included in 2.5 µL of 10X PCR buffer (FastSart Taq DNA Polymerase Kit, Roche); 200 µM of (0.5 µL at 10 mM) dNTP solution (EMD Millipore Corp, Burlington, MA, USA); 0.04 U (0.2 µL at 5 U/µL) of FastStart Taq DNA Polymerase (FastSart Taq DNA Polymerase Kit, Roche); 5 µL (20 ng) of genomic DNA (DNA extracts were diluted to standardize the DNA concentration at 4 ng/μL); and DNase-free water, up to a final reaction volume of 25 µL. 

Amplification was performed on a Gene Amp^®^ PCR System 9700 (Applied Biosystems; Thermo Fisher, Waltham, MA, USA). The PCR conditions were initial denaturation at 95 °C for 10 min, followed by 35 cycles of denaturation at 95 °C for 30 s, annealing at 55 °C for 30 s, extension at 72 °C for 1 min, and final extension at 72 °C for 10 min.

After purification through columns of the MinElute PCR Purification Kit (Qiagen Sciences Ing.), sequencing was performed by Sanger’s method (IMRB Genomics Platform). Sequences were analyzed using Bioedit (Hall, T.A. Version 7.2.5). Each sequence from our isolates was compared to the sequence of the type strains of each species within the *Flavi* section. Sequences used for comparison are based on the most recent taxonomic revision of the *Flavi* section published by Houbraken et al. in 2020 [41]. These sequences are presented in Table S2. Isolates were identified to a given species if the homologies with sequences of type strains were >98%. When there were mismatches between calmodulin and beta-tubulin, we used the most discriminating gene (between beta-tubulin and calmodulin) as defined by Houbraken et al. [41]. Phylogenetic trees of β-tubulin and calmodulin genes were built with maximum parsimony likelihood method with MEGA (Version 11.0.13) software. β-tubulin and calmodulin genes partial sequences were concatenated, and phylogenetic trees were also generated with these concatenated sequences. Using a heatmap, we labeled the tree obtained from the concatenation of calmodulin and β-tubulin with the MICs for each isolate.

### 2.3. Antifungal Susceptibility Testing

Isolates were revived from storage and subcultured on Sabouraud-agar slanted tubes. In vitro resistance to azoles was screened by subculturing each isolate on RPMI agar plates supplemented with itraconazole (Sigma-Aldrich, Saint-Quentin Fallavier, France) at 4 mg/L and voriconazole (Sigma-Aldrich) at 1 mg/L, as previously described [59]. After that, MICs for azoles were determined by EUCAST method.

The antifungal agents used in this study were amphotericin B (Sigma-Aldrich, Saint Quentin-Fallavier, France), itraconazole (Sigma-Aldrich), voriconazole (Pfizer Inc., New-York, NY, USA), isavuconazole (Basilea, Bâle, Suisse), posaconazole (Merck & Co Inc., Kenilworth, NJ, USA), and caspofungin (Merck & Co Inc.). The final concentrations tested ranged from 0.015 to 8 mg/L for each drug. Plates were incubated at 35 °C for 48 h in a humidified atmosphere. After incubation, the microplates were read with a reading mirror (for all drugs but caspofungin) to visualize the fungal growth in each well and determine the MIC as the lowest concentration with no visible growth. For caspofungin, an inverted microscope was used to determine the antifungal concentration that produced a visible change in the morphology of the hyphae compared with the growth control well (Minimum Effective Concentration). The EUCAST has set breakpoints for the interpretation of antifungal susceptibility testing results for itraconazole (Resistant isolates: MIC > 1 mg/L) and isavuconazole (Resistant isolates: MIC > 2 mg/L) [60]. For other antifungal drugs, breakpoints are not available, and we used ECOFFs for the interpretation: isolates were considered non-wild type when MIC was >4 mg/L for amphotericin B; >2 mg/L for voriconazole and >0.5 mg/L for posaconazole. Breakpoints and ECOFFs of echinocandins have not been set yet, and rates of resistance have not been calculated. *A. flavus* ATCC 204304, *Candida krusei* ATCC 6258, and *Candida parapsilosis* ATCC 22019 were used as quality control strains.

## 3. Results

### 3.1. Molecular Identification and Cryptic Species

Sequence analysis showed that 138 isolates were *A. flavus ss*, and two isolates were different cryptic species, *A. parasisticus/sojae* and *A. nomiae*. *A. flavus ss* sequences have been deposited in GenBank with accession numbers from OR285555 to OR285692 for beta-tubulin and accession numbers from OR226374 to OR266455 and from OR285498 to OR2885554 for calmodulin. For β-tubulin sequences, the phylogenetic trees built with 458 bp alignment length are shown in Figure S2. For calmodulin sequences, the phylogenetic trees built with 512 bp alignment length are shown in Figure S3. Among *A. flavus ss* isolates, 5 and 10 different sequence patterns were identified for β-tubulin and calmodulin, respectively (Tables S3 and S4).

The β-tubulin sequence (GenBank accession number OR455454) of isolate HEGP 1350 showed 99.7% and 97% homology with the reference sequences of *A. nomiae* and *A. pseudonomiae*, respectively. The calmodulin sequence (GenBank accession number OR455456) of isolate HEGP 1350 showed 98.6% and 99.1% homology with *A. nomiae and pseudonomiae* reference sequences, respectively. We conclude that this isolate is *A. nomiae*.

The β-tubulin sequence (GenBank access number OR455455) of isolate HEGP 3223 showed 99.2% and 100% homology with the reference sequences of *A. parasiticus* and *A. sojae*, respectively. The calmodulin sequence (GenBank access number OR455457) of isolate HEGP 3223 showed 100% homology with the reference sequences of *A. parasiticus* and *A. sojae*, respectively.

**Figure 1:**
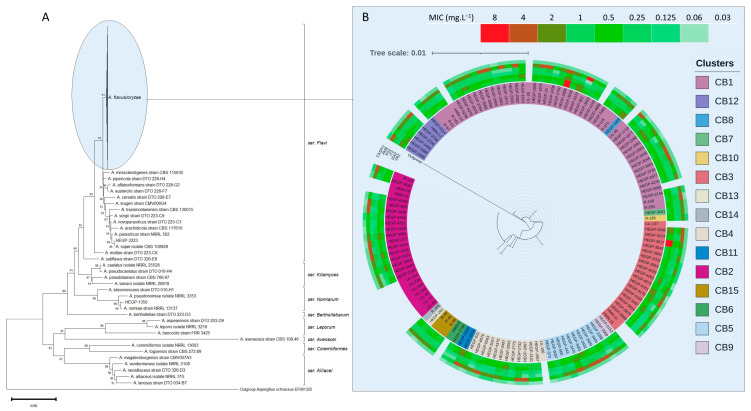
Phylogenetic tree of *A. flavus* of concatenated partial β-tubulin and calmodulin sequences. (**A**) All isolates and reference sequences. (**B**) Only *A. flavus ss* with *A. aflatoxiformans* as outgroup. The evolutionary history was inferred using the Neighbor-joining method [61]. The optimal tree is shown. The percentage of replicate trees in which the associated taxa clustered together in the bootstrap test (1000 replicates) are shown next to the branches [62]. The tree is drawn to scale, with branch lengths in the same units as those of the evolutionary distances used to infer the phylogenetic tree. The evolutionary distances were computed using the Maximum Composite Likelihood method [63] and are in the units of the number of base substitutions per site. This analysis involved 175 nucleotide sequences. All ambiguous positions were removed for each sequence pair (pairwise deletion option). There was a total of 970 positions in the final dataset. Evolutionary analyses were conducted in MEGA11 [64].

The phylogenetic tree of concatenated calmodulin and β-tubulin sequences is presented in Figure 1. Concatenated calmodulin and β-tubulin sequences showed 15 patterns (Table S5).

### 3.2. Antifungal Susceptibility Testing and Azole MICs Distribution

For logistical reasons, we were only able to determine MICs on 120 isolates from the HEGP hospital. Resistance screening revealed two voriconazole-resistant isolates collected in December 2005 and September 2007 among the 120 *A. flavus ss* isolates.

Table 1 and Table 2 show the distribution and values of MICs for the 120 *A. flavus ss* determined by the EUCAST method. For *A. flavus ss* isolates, the geometric mean MICs of voriconazole, isavuconazole, itraconazole, posaconazole, amphotericin B, and caspofungin were 0.78, 1.04, 0.25, 0.22, 1.90, and 0.06 mg/L, respectively. Of the 120 *A. flavus ss* isolates, 2 (1.67%) had isavuconazole MICs higher than clinical breakpoints. These two isolates were cross-resistant to voriconazole and isavuconazole with MICs of 8 mg/L for both drugs and were non-wild type for posaconazole with MICs of 1 mg/L. One of these two isolates had an itraconazole MIC of 2 mg/L, which can be interpreted as resistance to this drug. Of the remaining isolates, six others had posaconazole MICs of 1 mg/L, and one isolate had an amphotericin MIC of 8 mg/L. Table 3 shows MICs for *A. nomiae* and *A. parasiticus/sojae*. Geometric mean MICs were 0.50, 0.71, 0.35, 0.18, 2.83, and 0.04 mg/L for voriconazole, isavuconazole, itraconazole, posaconazole, amphotericin B, and caspofungin, respectively.

## 4. Discussion

In the present study, we report on the precise identification of a large collection of *Flavi* section isolates and their antifungal susceptibility. Given their importance in human, animal, and plant pathologies and their use in food and pharmaceutical industries, a better knowledge of the species of section *Flavi* is of prime importance. According to the last taxonomical revision, the *Flavi* section comprises 35 species grouped in eight series [41]. In the present study, most of the isolates were *A. flavus ss* (98.4%), showing that cryptic species seem to be rare in clinical samples. Within *A. flavus ss* isolates, calmodulin polymorphism was more marked than beta-tubulin polymorphism, as also observed by Frisvad [40]. The former led to the definition of 10 clusters, while the latter revealed 5 clusters. Concatenation of the two sequences revealed 15 clusters. Overlaying these clusters with susceptibility data revealed no specific profile for any given cluster. Based on calmodulin and beta-tubulin partial sequences, only two cryptic species were identified: *A. parasiticus/sojae* and *A. nomiae*. As the epidemiology of *Aspergillus* may depend on climatic conditions and the type of patients hospitalized, our results do not necessarily reflect the overall epidemiology in hospitals in France. Despite the mismatch between calmodulin and beta-tubulin for isolate HEGP 1350, since beta-tubulin is discriminating between *A. nomiae* and *A. pseudonomiae*, whereas calmodulin is not [40], we concluded that our isolate is *A. nomiae*. Neither beta-tubulin nor calmodulin discriminate between *A. parasiticus* and *A. sojae. A. sojae* is a domesticated nontoxigenic species associated with *A. parasiticus,* as *A. oryzae* is associated with *A. flavus* [40,65]. Several cases of infection due to cryptic species of section *Flavi* have been reported in the literature (Table 4). Some cases of *A. flavus ss* infection reported in France, with underlying disease, treatment, and outcome, are presented in Table S5. *A. parasiticus* and *A. nomiae* are two of the major aflatoxin-producing species. *A. nomiae* has been reported in cases of pulmonary disease [46,66,67,68], onychomycosis [69], keratitis [6,70], and rhinofacial aspergillosis [71]. In our study, both species were recovered from pulmonary specimens but were considered colonizers. Among the other cryptic species, *A. pseudonomiae* has been reported in a sinus tissue sample from a patient with fungal rhinosinusitis [72] and in keratitis [73]. *A. alliaceus, A. caelatus, A. minisclerotigenes, A. novoparasiticus*, *A. tamarii*, *A. pseudotamarii*, and *A. oryzae* var. *effusus* are the other cryptic species that have been isolated from clinical samples. *A. tamarii* has been reported as a cause of primary cutaneous aspergillosis [74,75], wound infection [72,76,77], onychomycosis [78], keratitis [6,7,8,9,10,79], and invasive nasosinusal aspergillosis [80,81]. *A. pseudotamarii* could also cause keratitis [11]. *A. alliaceus* is an etiological agent of invasive pulmonary aspergillosis [46,82,83], and this species produces ochratoxin [84]. *A. oryzae* var. *effusus* has been reported as a cause of keratitis [6] and has been isolated from the lower respiratory tract of hospitalized patients [85]. In 2019, one case of *A. caelatus* airway colonization in a chronic obstructive pulmonary disease patient was reported in Colombia [86]. *A. minisclerotigenes* has been reported as a cause of sinus [45] infection and keratitis [87]. *A. novoparasiticus* has been isolated from the sputum of a patient in São Paulo, Brazil [88]. The low number of clinical cases could be explained by an underestimation of cryptic species due to their difficulty in identification. Although molecular sequencing of β-tubulin/calmodulin remains the gold standard for a precise species identification, new technologies such as whole-genome sequencing are now used for taxonomic purposes [89]. Other molecular techniques are also available for assessing the genetic diversity among *A. flavus* isolates [90]. Mass spectrometry may be interesting in routine clinical microbiology laboratories [91]. Environment plays an important role in the epidemiology of *A. flavus* human infection. Infections due to this species are more prevalent in dry and hot climatic regions, and some clinical cases reported in France are imported infections contracted in Africa [24,25,32]. Infections are mostly acquired by exposure to *A. flavus* spores in the environment, and nosocomial infections in hospitals have also been reported from surgery unit air and surface [30,92].

Among our isolates, two resistant isolates (1.67%) with voriconazole and isavuconazole MICs of 8 mg/L recovered from patients with long-term treatment with voriconazole were detected. This observation is in favor of the selection for cross-resistance to voriconazole and isavuconazole by antifungal pressure during the patient’s treatment with voriconazole. The first clinical isolate of voriconazole-resistant *A. flavus* has been recovered from the lung biopsy of a Chinese patient who had also previously been treated with voriconazole [53]. Voriconazole-resistant isolates have been reported from China [53], India [54,55,93], South Korea [56], and Spain [94,95], with a prevalence between 2.5 and 14% among clinical isolates. One of our isolates showed amphotericin MIC at 8 mg/L. Cases of resistance to amphotericin B have also been reported, and the MICs of amphotericin B for *A. flavus* are generally higher than for *A. fumigatus* [96]. In the present study, none of the azole-resistant isolates and isolates with MICs of 8 mg/L for amphotericin B were involved in invasive aspergillosis. 

The emergence of resistant isolates highlights the importance of antifungal susceptibility testing, particularly for patients undergoing long-term treatment. The best techniques are the microdilution broth reference techniques (EUCAST or CLSI), but other techniques, such as the concentration gradient strip (CGS) method, can also be used. Indeed, it has been shown that the results obtained with the CGS method correlated well with those obtained with the reference techniques for *A. flavus* [97].

## 5. Conclusions

This study demonstrated that cryptic species within the *Flavi* section and azole-resistant *A. flavus ss* can be found among clinical isolates. Therefore, precise identification to the species level and routine antifungal susceptibility testing are needed.

## Figures and Tables

**Table 1:** MIC distribution of 6 antifungal drugs against 120 *A. flavus ss* isolates.

Antifungal Drugs	Number of Isolates for the Following MIC (mg/L)									
	0.015	0.03	0.06	0.125	0.25	0.5	1	2	4	8
VRZ	-	-	-	-	-	58	53	7	-	**2**
ISA	-	-	-	-	-	19	81	18	-	**2**
ITZ	-	-	-	29	64	23	3	**1**	-	-
PSZ	-	1	15	24	54	18	**8**		-	-
AMB	-	-	-	-	1	2	34	51	31	**1**
CAS	-	20	83	17	-	-	-	-	-	-

VRZ: voriconazole; ISA: isavuconazole; ITZ: itraconazole; PSZ: posaconazole; AMB: amphotericin B; CAS: caspofungin. Resistant isolates (MIC > 1 mg/L for ITZ, MIC > 2 mg/L for ISA) and non-wild-type isolates (MIC was >4 mg/L for AMB, >2 mg/L for VRZ, and >0.5 mg/L for PSZ) are indicated in bold.

**Table 2:** Statistical indices of the distribution of MICs for 6 antifungal drugs against 120 *A. flavus* ss isolates.

Indice (mg/L)	Antifungal Drug (mg/L)					
	VRZ	ISA	ITZ	PSZ	AMB	CAS
Min	0.5	0.5	0.125	0.031	0.25	0.03
Max	8	8	2	1	8	0.125
GMean	0.77	1.03	0.25	0.22	1.91	0.06
MIC_50_	1	1	0.25	0.25	2	0.06
MIC_90_	1	2	0.5	0.5	4	0.125

VRZ: voriconazole; ISA: isavuconazole; ITZ: itraconazole; PSZ: posaconazole; AMB: amphotericin B; CAS: caspofungin; Min: minimum; Max: maximum; GMean: geometric mean; MIC_50_: minimal inhibitory concentration that inhibits 50% of the isolates; MIC_90_: minimal inhibitory concentration that inhibits 90% of the isolates.

**Table 3:** MICs for the two cryptic species.

Species	MIC (mg/L)					
	VRZ	ISA	ITZ	PSZ	AMB	CAS
*A. parasiticus*	0.5	0.5	0.25	0.125	2	0.06
*A. nomiae*	0.5	1	0.5	0.25	4	0.03

MICs were determined by the EUCAST method. VRZ: voriconazole; ISA: isavuconazole; ITZ: itraconazole; PSZ: posaconazole; AMB: amphotericin B; CAS: caspofungin.

**Table 4:** *Flavi* section cryptic species in the literature.

Species	Localization of Infection	Treatment		Outcome	Reference
		Local	Systemic		
*A. alliaceus*	Lung	None	VRZ	Survived	[46]
*A. alliaceus*	Lung	None	VRZ	Death	[82]
*A. alliaceus*	Lung	None	AMB + CAS, then ITZ	Death	[83]
*A. caelatus*	Lung (colonization)	none	none	NA	[86]
*A. minisclerotigenes*	Sinus	NA	NA	NA	[45]
*A. minisclerotigenes*	Paranasal	NA	NA	NA	[45]
*A. minisclerotigenes*	Eye	AMB + VRZ	FCZ	Corneal transplant	[87]
*A. novoparasiticus*	Lung	NA	NA	NA	[88]
*A. novoparasiticus*	Lung	NA	NA	NA	[88]
*A. nomiae*	Lung	none	AMB + ITZ	Death	[66]
*A. nomiae*	Lung	none	ITZ	Survived	[66]
*A. nomiae*	Corneal	NTM, ECZ, ITZ, KTZ	none	Corneal perforation, scleral extension	[70]
*A. nomiae*	Nail	Amorolfine	ITZ	Cured	[69]
*A. nomiae*	Sputum	VRZ	none	Death	[67]
*A. nomiae*	Lung	AMB nebulized	ISA + Anidulafungin	Death	[46]
*A. nomiae*	Skin	none	AMB	Death	[71]
*A. nomiae*	Lung	none	CAS + PSZ + bilobectomy	Cured	[68]
*A. nomiae*	Eye	NA	NA		[73]
*A. oryzea* var. *effusus*	Eye	none	none	Tectonic KP, vitrectomy	[6]
*A. pseudonomiae*	Sinus tissue	surgery		Cured	[72]
*A. pseudonomiae*	Eye	NA		NA	[73]
*A.pseudonomiae*	Eye				[7]
*A. pseudotamarii*	Eye	NTM; ITZ	ITZ	Improved	[11]
*A. tamarii*	Eye	NTM, ECZ (then CLT), ITZ	KTZ	Good response, no follow-up	[10]
*A. tamarii*	Eye	NTM, ECZ (then CLT), ITZ	KTZ	Healed, no follow-up	[10]
*A. tamarii*	Eye	NTM, ECZ, ITZ, AMB	KTZ	Evisceration	[10]
*A. tamarii*	Eye	NTM, ITZ	KTZ	Healed	[10]
*A. tamarii*	Eye	NTM, ECZ, ITZ	KTZ	Healed	[10]
*A. tamarii*	Eye	NTM, ECZ, ITZ	KTZ	No response, no follow-up	[10]
*A. tamarii*	Eye	NTM, ECZ, FCZ	KTZ	Improved, central nebular scar	[9]
*A. tamarii*	Eye	VRZ, AMB, NTM	AMB, VRZ	Improved, extensive corneal scar	[8]
*A. tamarii*	Eye	VRZ	ITZ	Improved, no surgery	[6]
*A. tamarii*	Eye	VRZ after surgery	none	Tectonic KP, vitrectomy	[6]
*A. tamarii*	Eye	VRZ after surgery	none	Tectonic KP, vitrectomy	[6]
*A. tamarii*	Eye	VRZ	none	Tectonic KP, OKP, intraocular lens	[6]
*A. tamarii*	Skin	CLT	AMB iv	Resolving of cutaneous lesions	[74]
*A. tamarii*	Skin	none	ITZ	Complete recovery	[75]
*A. tamarii*	Nasosinusal		ITZ and surgery	Excellent local results at a follow-up of one year	[80]
*A. tamarii*	Sputum (colonization)	none	none	Survived	[66]
*A. tamarii*	Nail	Urea cream, Terbinafine		Cured	[78]
*A. tamarii*	Corneal	NA	NA	NA	[7]
*A. tamarii*	Eye	NTM	ITZ	Vascularized corneal opacity	[79]
*A. tamarii*	Tissue (RTA)		AMB		[72]
*A. tamarii*	Skin	AMB	VRZ then AMB	Survived	[76]
*A. tamarii*	Left foot biopsy	NA	NA	NA	[77]

KTZ: Ketoconazole; ECZ: Econazole; NTM: Natamycin; VRZ: Voriconazole; FCZ: Fluconazole; ITZ: Itraconazole; PSZ: Posaconazole; AMB: Amphotericin B; CLT: Clotrimazole; Ref: reference.

## Data Availability

All MIC data are presented within the manuscript.

## Supplementary Materials

The following supporting information can be downloaded at https://www.mdpi.com/article/10.3390/microorganisms11102429/s1. Table S1: List of isolates with origin of samples, diagnosis, clinical context; Table S2: Reference strains list; Table S3: Variability of β-tubulin sequences (β-tubulin and calmodulin); Table S4: Variability of calmodulin sequences; Table S5: Sequence types after concatenation of calmodulin and β-tubulin; Table S6: Cases of *A. flavus* ss infection reported in France; Figure S1: Origin of samples; Figure S2: Phylogenetic tree of *A. flavus* partial β-tubulin sequences; Figure S3: Phylogenetic tree of *A. flavus* partial calmodulin sequences.
