# Supplementary material for: The Molecular Identification and Antifungal Susceptibility of Clinical Isolates of Aspergillus Section Flavi from Three French Hospitals
Source: Microorganisms. 2023 Sep 28;11(10):2429. doi: 10.3390/microorganisms11102429 (PMC10609271; doi:10.3390/microorganisms11102429)

microorganisms

MDPI

Supplementary materials

Table S1: List of isolates with origin of samples, diagnosis, clinical context; All isolates are *A. flavus* ss except *A. nomiae* and *A. parasiticus/sojae* which are marked in bold

Isolate	Sample	Diagnosis	Clinical context
HEGP-282	Sputum	Colonization	Lung transplant
HEGP-362	BA	Colonization	Throat cancer
HEGP-377	BA	Invasive pulmonary aspergillosis	Hematologic malignancy
HEGP-443	Sputum	Colonization	Pneumectomy for mycobacteriosis
HEGP-483	Sputum	Colonization	Corticosteroid therapy in a patient with chronic hepatitis
HEGP-560	Biopsie	Colonization	Pneumectomy for mycobacteriosis
HEGP-573	Biopsie	Colonization	Pneumectomy for mycobacteriosis
HEGP-631	Sputum	Colonization	Kidney transplant patient on corticosteroids
**HEGP-1350[(1)]**	**BA**	**Colonization**	**Pre-kidney transplant assessment for hepatorenal polycystosis**
HEGP-1699	Sputum	Colonization	Lung transplant for cystic fibrosis
HEGP-1719	Sputum	Colonization	COPD and DDB in patients with IgG 3 deficiency
HEGP-1816	BA	Colonization	HTA in patient with vascular prosthesis for aortic rupture
HEGP-1875	Sputum	Colonization	Lung transplant
HEGP-1877	Sputum	Colonization	DDB on tuberculosis sequelae
HEGP-1967	Sputum	Colonization	Asthma
HEGP-2082	Sinus	Sinusitis	Hematologic malignancy
HEGP-2101	Sputum	Colonization	COPD and DDB in patients with IgG 3 deficiency
HEGP-2160	BA	Colonization	Stroke and bacterial septic shock
HEGP-2282	Sputum	Colonization	COPD and DDB in patients with IgG 3 deficiency
HEGP-2288	Biopsie	Colonization	Digestive sprue and sinusitis
HEGP-2325	Sputum	Invasive pulmonary aspergillosis	Hematologic malignancy
HEGP-2335	BA	Colonization	Lung cancer
HEGP-2436	BAL	Colonization	Lung cancer
HEGP-2496	BA	Colonization	Lung transplant
HEGP-2661	Sputum	Colonization	Lung transplant for cystic fibrosis
HEGP-2667	BA	Invasive pulmonary aspergillosis	Bone marrow transplant
HEGP-2671	BAL	Colonization	Lung transplant for cystic fibrosis
HEGP-2732	BAL	Invasive pulmonary aspergillosis	Bone marrow transplant
HEGP-2750	BA	Colonization	Lung transplant
HEGP-2770	BA	Invasive pulmonary aspergillosis	Bone marrow transplant
HEGP-2798	BA	Colonization	Lung transplant for cystic fibrosis
HEGP-2803	BA	Colonization	Lung transplant
HEGP-2845	BA	Colonization	Lung transplant
HEGP-2855	BAL	Colonization	Lung transplant
HEGP-2862	Brosse	Colonization	Lung transplant
HEGP-2885	Sputum	Colonization	Lung transplant
HEGP-2930	Sputum	Invasive pulmonary aspergillosis	Lung transplant for cystic fibrosis
HEGP-2998	Biopsie	Aspergilloma	System disorder under corticosteroid therapy
HEGP-3005	Sputum	Colonization	Hemoptysis and dyspnea in patient with a history of tuberculosis
HEGP-3036	BA	Colonization	Lung transplant for cystic fibrosis
HEGP-3041	Sputum	Colonization	Lung transplant
HEGP-3048	BA	Colonization	Lung transplant for cystic fibrosis
HEGP-3056	BA	Colonization	Epiglottis cancer
HEGP-3131	Arterial biopsy	Endovascular infection	Popliteal aneurysm and thrombosis with lower limb ischemia
HEGP-3153	BAL	Colonization	Lung transplant
HEGP-3161	Arterial biopsy	Endovascular infection	Popliteal aneurysm and thrombosis with lower limb ischemia
HEGP-3214	BAL	Colonization	Lung transplant

**HEGP-3223** (2)	**Sputum**	**Colonization**	**B lymphoma, breast cancer, type 2 diabetes and follow-up of pulmonary aspergillosis due to *A. terreus***
HEGP-3353	Sputum	Colonization	Bacterial pneumonia
HEGP-3370	BA	Invasive pulmonary aspergillosis	COPD under Corticosteroid therapy
HEGP-3386	BAL	Colonization	Lung transplant
HEGP-3399	Vegetation	Endocarditis	Idiopathic aplasia
HEGP-3401	BA	Colonization	Hematologic malignancy
HEGP-3403	Sputum	Colonization	Lung transplant
HEGP-3409	BA	Colonization	Lung transplant
HEGP-3419	Oreille	External otitis	NA
HEGP-3432	Sputum	Colonization	Lung transplant
HEGP-3463	Biopsie	Nasal aspergillosis	Heroin addict
HEGP-3470	BA	Invasive pulmonary aspergillosis	Hematologic malignancy
HEGP-3480	BA	Colonization	Lung transplant
HEGP-3497	BA	Colonization	Lung cancer
HEGP-3517	Sputum	Colonization	Breast cancer
HEGP-3543	Sputum	Colonization	Lung transplant for cystic fibrosis
HEGP-3556	Sputum	Colonization	Lung transplant for emphysema
HEGP-3568	Sputum	Colonization	Cardiac transplant
HEGP-3661	BAL	Colonization	Lung transplant for emphysema
HEGP-3702	BA	Colonization	Lung transplant for emphysema
HEGP-3706	Sinus	Sinusitis	Hematologic malignancy
HEGP-3733	BA	Colonization	Lung transplant for cystic fibrosis
HEGP-3784	BAL	Colonization	Hematologic malignancy
HEGP-3799	BAL	Colonization	Cystic fibrosis
HEGP-3818	BA	Colonization	Lung transplant for cystic fibrosis
HEGP-3918	Oreille	Chronic otitis	NA
HEGP-3969	Oreille	External otitis	NA
HEGP-3977	BAL	Colonization	Lung transplant
HEGP-3978	BA	Colonization	Lung transplant
HEGP-4029	Oreille	External otitis	Cholesteatoma
HEGP-4061	Oreille	External otitis	Cholesteatoma
HEGP-4065	Sputum	Colonization	Lung transplant
HEGP-4073	Sputum	Colonization	Vasculitis (Wegener's disease) treated with immunosuppressants
HEGP-4108	BA	Colonization	Lung transplant
HEGP-4114	Oreille	External otitis	Cholesteatoma
HEGP-4150	Sputum	Colonization	Cardiac transplant
HEGP-4161	Oreille	External otitis	Tympanoplasty
HEGP-4175	Oreille	External otitis	Tympanoplasty
HEGP-4189	BA	Colonization	COPD
HEGP-4245	BA	Colonization	Lung transplant
HEGP-4251	BA	Colonization	Lung cancer with lobectomy
HEGP-4254	Oreille	External otitis	NA
HEGP-4370	BA	Bronchopulmonary aspergillosis	Lung cancer
HEGP-4378	Sputum	Colonization	Sarcoidosis
HEGP-4436	BA	Colonization	Lung transplant for cystic fibrosis
HEGP-4888	BA	Colonization	Diffuse bronchial dilatation
HEGP-5027		NA	NA
HEGP-5065	Sputum	Colonization	Asthma and COPD
HEGP-5238	BAL	Colonization	Asthma and Diffuse bronchial dilatation
HEGP-5407	Biopsie	Invasive aspergillosis of the arm	Road accident
HEGP-5481	Sputum	Invasive pulmonary aspergillosis	Lung transplant
HEGP-5744	Liquide de dialyse	NA	Chronic kidney disease
HEGP-5816	Oreille	External otitis	Tympanoplasty
HEGP-5875	Sinus	External otitis	Tympanoplasty
HEGP-5899	Oreille	External otitis	Tympanoplasty

HEGP-6063	BA	Colonization	Lung transplant for amylose
HEGP-6070	BA	Colonization	Lung transplant for amylose
HEGP-6097	BAL	Allergic bronchopulmonary aspergillosis	Asthma and Diffuse bronchial dilatation
HEGP-6099	BA	Allergic bronchopulmonary aspergillosis	Asthma and Diffuse bronchial dilatation
HEGP-6153	Sputum	Colonization	Smoking-induced COPD
R057	BA	Colonization	Lung cancer
R275	BA	Colonization	Smoking-induced COPD
HEGP 022	BA	Colonization	Diffuse bronchomalacy
R284	BA	Colonization	Kidney cancer
R38	Sputum	Colonization	Smoking-induced COPD and HIV
R44	Sputum	Colonization	NA
R46	Sputum	Colonization	NA
R51	Sputum	Colonization	Asthma and emphysema
R80	Sputum	NA	NA
R110	Sputum	Colonization	NA
R118	Ongle	NA	NA
R158	Sinus	NA	Diabetes and history of tuberculosis
R161	Sinus	NA	Diabetes and history of tuberculosis
R185	Sputum	Colonization	Lung transplant for cystic fibrosis
R186	BAL		Colonization
LIL-005	Sputum	Colonization	Cystic Fibrosis
LIL-028	Sputum	Colonization	Cystic Fibrosis
LIL-066	Sputum	Colonization	Cystic Fibrosis
LIL-070	Sputum	Colonization	Cystic Fibrosis
LIL-082	Sputum	Colonization	Cystic Fibrosis
LIL-108	BA	Colonization	COPD
LIL-137	BA	Colonization	Ingestion of caustic
LIL-154	Sputum	Allergic bronchopulmonary aspergillosis	Cystic Fibrosis
LIL-156	Sputum	Allergic bronchopulmonary aspergillosis	Cystic Fibrosis
LIL-164	Sinus	Colonization	Chronic sinusitis
LIL-166	Sputum	Colonization	Cystic Fibrosis
LIL-170	Sputum	Allergic bronchopulmonary aspergillosis	Cystic Fibrosis
LIL-226	Sputum	Colonization	Cystic Fibrosis
HMN – 8	Hallux biospsy	Infection	Hematologic malignancy
HMN - 11	BA	NA	NA
HMN - 12	BAL	Colonization	Iatrogenic pneumonitis in liver transplant recipients
HMN - 14	BA	NA	NA
HMN - 15	BAL	NA	NA
HMN - 17	Nasal	NA	NA
HMN - 19	Sputum	NA	NA

[1]***A. nomiae;*** [2]***A. parasiticus/sojae***; BA: Bronchial aspiration; BAL: Broncho-Alveolar Lavage; NA: not available

Table S2: Reference strains list

Species	Strain number	Sequence accession number (GenBank)	
		Calmodulin	β-tubulin
Aspergillus aflatoxiformans	DTO 228-G2	MG518076.1	MG517706.1
Aspergillus alliaceus	NRRL 315	EF661534.1	EF661465.1
Aspergillus arachidicola	CBS 117610	EF202049.1	EF203158.1
Aspergillus aspearensis	DTO 203-D9	MG518040.1	MG517669.1
Aspergillus austwickii	DTO 228-F7	MG518072.1	MG517702.1
Aspergillus avenaceus	NRRL 517	FJ491496.1	FJ491481.1
Aspergillus bertholletiae	DTO 223-D3	KY924678.1	MG517689.1
Aspergillus caelatus	NRRL 25528	MG518018.1	EF661470.1
Aspergillus cerealis	DTO 228-E7	MG518063.1	MG517693.1
Aspergillus coremiiformis	NRRL 13603	FJ491488.1	EU014104.1
Aspergillus flavus	NRRL 1957	EF661508.1	EF661485.1
Aspergillus hancockii	FRR 3425	MT211765.1	MT211764.1
Aspergillus krugeri	CMV006G4	MK451517.1	MK451098.1
Aspergillus lanosus	DTO 034-B7	MG518017.1	MG517633.1
Aspergillus leporis	NRRL 3216	EF661541	EF661499.1
Aspergillus luteovirescens	DTO 010-H1	MG517998.1	MG517625.1
Aspergillus magaliesburgensis	CMV007A3	MK451511.1	MK451116.1
Aspergillus minisclerotigenes	CBS 115635	MG518009.1	EF203148.1
Aspergillus mottae	DTO 223-C8	MG518058.1	HM803086.1
Aspergillus neoalliaceus	DTO 326-D3	MG518133.1	MG517763.1
Aspergillus nomiae	NRRL 13137	EF661531.1	AF255067.1
Aspergillus novoparasiticus	DTO 223-C3	MG518055.1	MG517684.1
Aspergillus oryzae	NRRL 447	EF661506.1	EF661483.1
Aspergillus parasiticus	NRRL 502	EF661516.1	EF661481.1
Aspergillus pipericola	DTO 228-H4	MG518087.1	MG517717.1
Aspergillus pseudocaelatus	DTO 010-H4	MG517995.1	MG517626.1
Aspergillus pseudonomiae	NRRL 3353	EF661529.1	EF661495.1
Aspergillus pseudotamarii	NRRL 25517	EF661521.1	EF203125.1
Aspergillus sergii	DTO 223-C9	MG518059.1	MG517688.1
Aspergillus sojae	CBS 100928	EF202041.1	KJ175494.1
Aspergillus subflavus	DTO 326-E8	MG518143.1	MG517773.1
Aspergillus tamarii	NRRL 20818	EF661526.1	EF661474.1
Aspergillus togoensis	CBS 272.89	FJ491489.1	FJ491477.1
Aspergillus transmontanensis	CBS 130015	HQ340083.1	HM803087
Aspergillus vandermerwei	NRRL 5108	EF661540.1	EF661469.1

Table S3: Variability of β-tubulin sequences

	Position in alignment			Number
	281	311	339	of isolates
Type 1	C	G	G	124
Type 2	.	A	.	2
Type 3	A	.	.	8
Type 4	T	.	.	1
Type 5	.	.	.	3

Table S4: Variability of calmodulin sequences

	Position in alignment										Number
	28	34	62	84	97	101	108	383	413	416	of isolates
Type 1	G	T	T	T	T	A	T	T	C	C	55
Type 2	.	.	.	_	A	.	.	G	.	T	26
Type 3	.	.	.	.	.	.	C	G	.	.	20
Type 4	.	.	.	_	A	.	.	G	.	.	16
Type 5	.	_	.	.	.	.	C	G	.	.	10
Type 6	T	.	.	_	A	.	.	G	.	.	5
Type 7	.	.	.	.	.	G	.	.	.	.	1
Type 8	.	.	.	.	.	.	.	.	A	.	1
Type 9	.	.	C	.	.	.	C	G	.	.	2
Type 10	.	.	.	.	Y	.	C	G	.	.	1

Table S5: Sequence types after concatenation of calmodulin and β-tubulin

		β-tubulin sequences types					Number of isolates per calmodulin types
		Type 1	Type 2	Type 3	Type 4	Type 5	
Calmodulin sequences types	Type 1	CB_1: 48		CB_{12}: 7			55
	Type 2	CB_2: 25			CB_{14}: 1		26
	Type 3	CB_3: 20					20
	Type 4	CB_4: 13	CB_{11}: 2	CB_{13}: 1			16
	Type 5	CB_5: 10					10
	Type 6	CB_6: 2				CB_{15}: 3	5
	Type 7	CB_7: 1					1
	Type 8	CB_8: 1					1
	Type 9	CB_9: 2					2
	Type 10	CB_{10}: 1					1
Number of isolates per β-tubulin types		123	2	8	1	3	137

CB_X: Calmodulin and β-tubulin concatenation sequences type x

Table S6: cases reported in France of *A. flavus ss* infection;

Clinical form	Treatment	Outcome	Underlying disease	Ref
Malignant external otitis	VRZ	Death	Diabetes	[12]
Malignant external otitis	VRZ	Improvement	Diabetes	[13]
Malignant external otitis (1 ; 2 and 9 cases)	VRZ	Survived but sequelae	Diabetes	[14]
Malignant external otitis	VRZ	Recovered	Diabetes	[15]
Pulmonary IA	VRZ	Improvement	HIV and PNC	[19]
Cerebral abscesses	none	Death	Amebiasis	[24]
Pulmonary IA	ISA then AMB	Death	Acute myeloid leukemia	[20]
Myositis	AMB then ITZ then VRZ	Healing	HIV	[25]
Pulmonary IA (3 cases)	NA	NA	Hematological Malignancies	[21]
Pulmonary IA and secondarary cutaneous IA	VRZ and CAS	Alive	Acute lymphoblastic leukemia	[3]
Pulmonary IA and secondarary cutaneous IA	VRZ	Death	Chronic lymphocytic leukemia	[3]
Primary cutaneous IA	VRZ and surgery	Alive	Acute myelogenous leukemia	[3]
Primary cutaneous IA	VRZ	Alive	Acute myelogenous leukemia	[3]
Primary cutaneous IA	VRZ and surgery	Alive	Acute myelogenous leukemia	[3]
Primary cutaneous IA	AMB and CAS	Alive	Acute lymphogenic leukemia	[3]
Primary cutaneous IA	VRZ	Alive	Heart transplantation	[3]
Malignant external otitis	VRZ		Diabetes and renal cell carcinoma	[16]

Endocarditis	AMB switched to CAS and VRZ	Favourable evolution	Heart valvular surgery for aortic valve insufficiency and thoracic aortic aneurysm	[30]
Endocarditis	AMB and 5FC	Death	Heart valvular surgery for aortic valve insufficiency	[30]
Endocarditis	CAS then AMB and VRZ	Death	Heart valvular surgery for aortic insufficiency	[30]
Spondylodiscitis	ITZ			[28]
Spondylodiscitis	VRZ	Favourable evolution	Diabetes	[29]
Chronic rhinosinusitis (3 cases)				[23]
Mitral valve endocarditis (after lung abscess)			Acute lymphoblastic leukemia	[31]
Bulky mediastinal aspergillosis	VRZ	Resolution	none	[32]
Hot tub pneumonitis	none	Favourable evolution	none	[35]
Rhinosinusitis	NA	NA	NA	[34]
Invasive aspergillosis (10 cases)	NA	NA	NA	[22]

VRZ: Voriconazole; ITZ: Itraconazole; PSZ: Posaconazole; AMB: Amphotericin B; CLT: Clotrimazole; NA: not available; CAS: Caspofungin; 5FC: Flucytosine; Ref: reference.

Figure S1: Origin of samples. Deep respiratory samples include: biopsy, brushing, bronchoalveolar lavage and bronchial aspiration; Other samples include: artery biopsies, dialysis fluid, nail, arm biopsy, hallux biopsy, endocardial biopsy

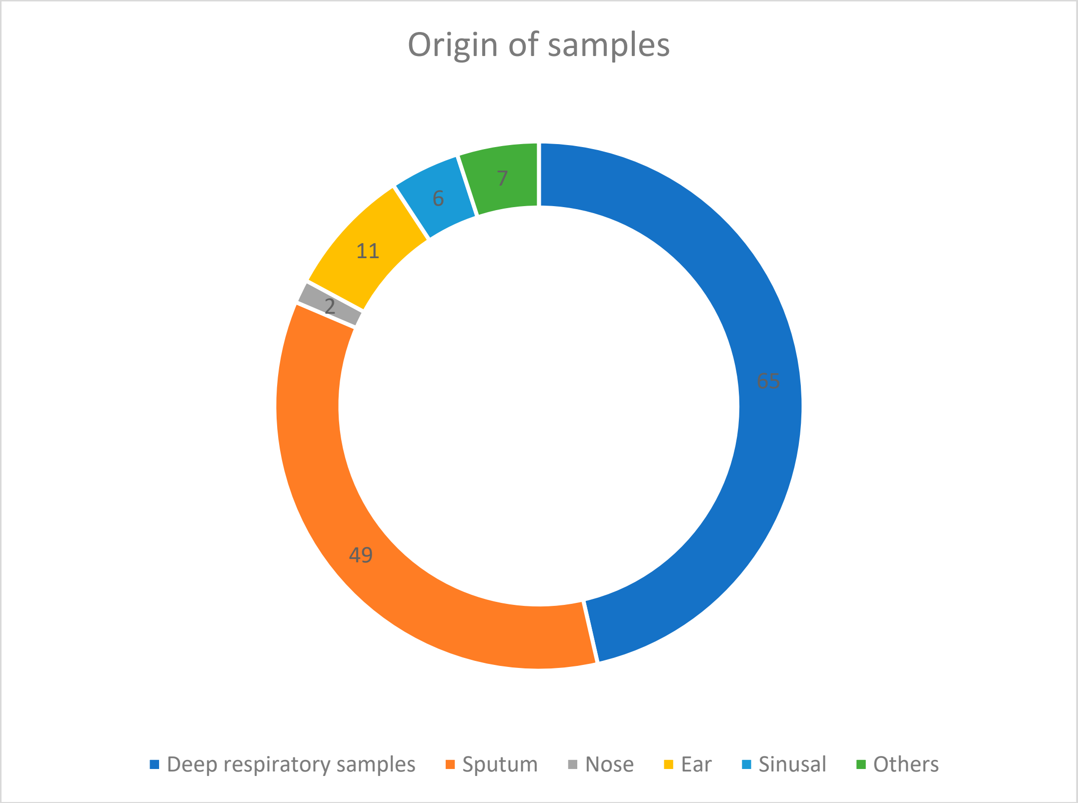

Figure S2: Phylogenetic tree of *A. flavus* partial β-tubulin sequences. The evolutionary history was inferred using the Neighbor-Joining method [59]. The optimal tree is shown. The percentage of replicate trees in which the associated taxa clustered together in the bootstrap test (1000 replicates) are shown next to the branches [60]. The tree is drawn to scale, with branch lengths in the same units as those of the evolutionary distances used to infer the phylogenetic tree. The evolutionary distances were computed using the Maximum Composite Likelihood method [61] and are in the units of the number of base substitutions per site. This analysis involved 175 nucleotide sequences. All ambiguous positions were removed for each sequence pair (pairwise deletion option). There were a total of 458 positions in the final dataset. Evolutionary analyses were conducted in MEGA11 [62]

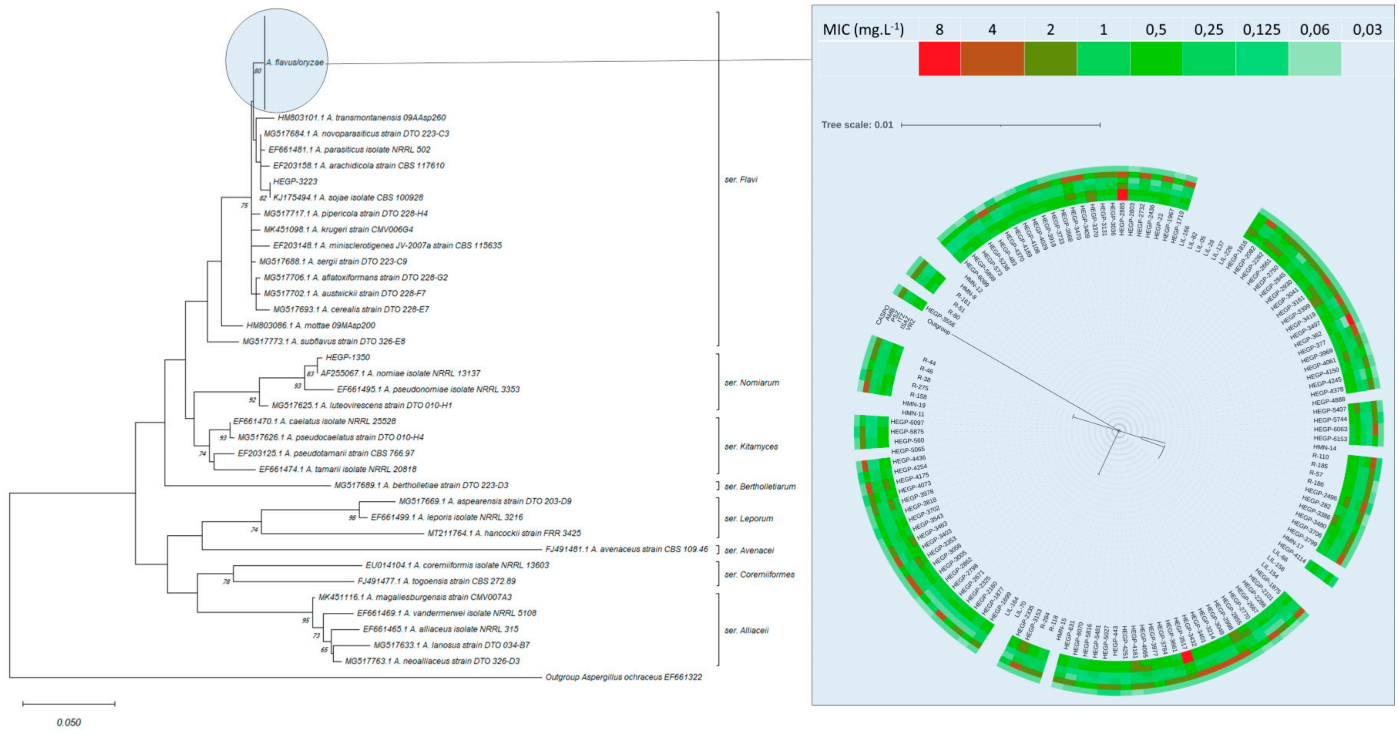

Figure S3: Phylogenetic tree of *A. flavus* partial calmodulin sequences. The evolutionary history was inferred using the Neighbor-Joining method [59]. The optimal tree is shown. The percentage of replicate trees in which the associated taxa clustered together in the bootstrap test (1000 replicates) are shown next to the branches [60]. The tree is drawn to scale, with branch lengths in the same units as those of the evolutionary distances used to infer the phylogenetic tree. The evolutionary distances were computed using the Maximum Composite Likelihood method [61] and are in the units of the number of base substitutions per site. This analysis involved 175 nucleotide sequences. All ambiguous positions were removed for each sequence pair (pairwise deletion option). There were a total of 542 positions in the final dataset. Evolutionary analyses were conducted in MEGA11 [62]

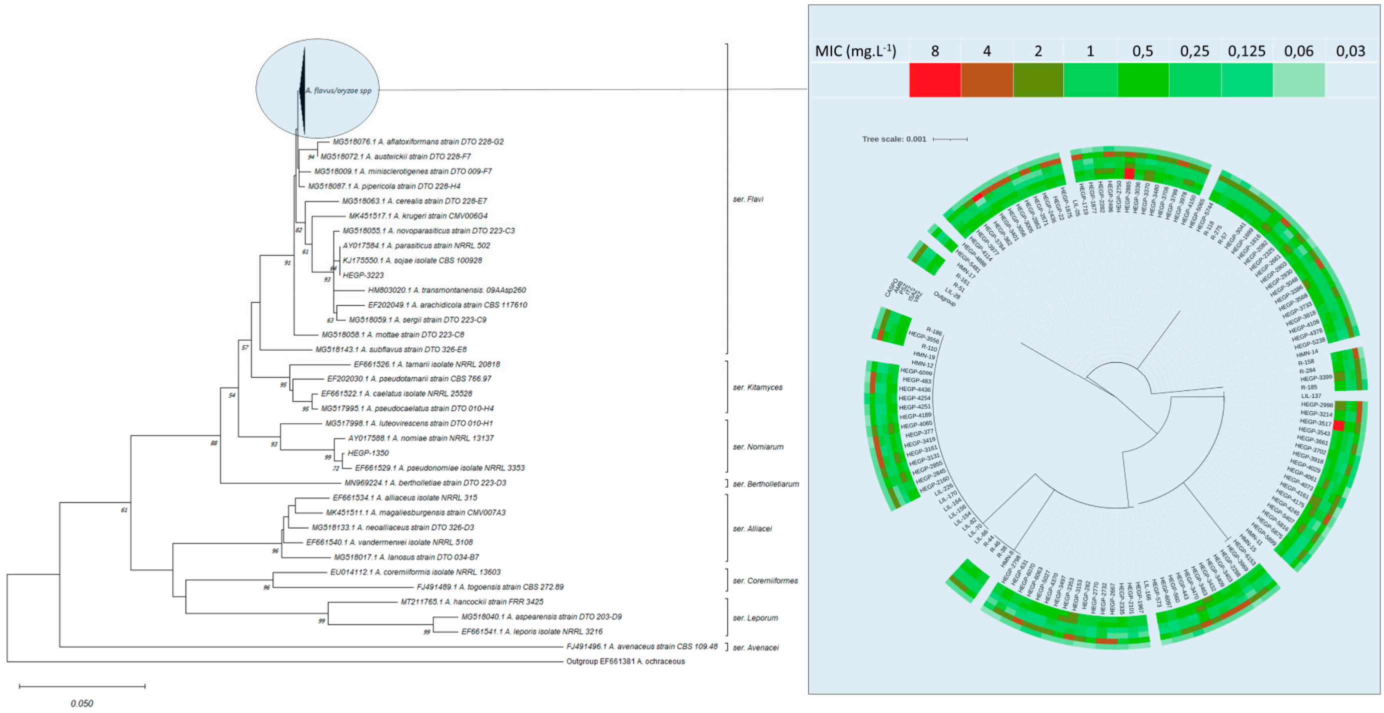

Supplement: Supplementary file 1 [file microorganisms-11-02429-s001.zip › microorganisms-2626088-supplementary.pdf]
